# Understanding the effects of dietary components on the gut microbiome and human health

**DOI:** 10.1007/s10068-020-00811-w

**Published:** 2020-08-29

**Authors:** Bryna Rackerby, Hyun Jung Kim, David C. Dallas, Si Hong Park

**Affiliations:** 1 Department of Food Science and Technology, Oregon State University, Corvallis, OR 97331 USA; 2 Korea Food Research Institute, Wanju, Jeollabuk-do 55365 South Korea; 3 School of Biological and Population Health Sciences, Nutrition, Oregon State University, Corvallis, OR 97331 USA

**Keywords:** Diet, Gut microbiome, Macronutrient, Micronutrient, Health

## Abstract

The gut microbiome is the complex microbial ecosystem found in the gastrointestinal tract of humans and animals. It plays a vital role in host development, physiology and metabolism, and has been implicated as a factor in brain function, behavior, mental health, and many disease states. While many factors, including host genetics and environmental factors, contribute to the composition of the gut microbiome, diet plays a large role. Microorganisms differ in their nutrient requirements, and alterations in host dietary composition can have strong impacts on the microbial inhabitants of the gastrointestinal tract. The health implications of these dietary and microbial changes are relevant as various global populations consume diets comprised of different macronutrient ratios, and many diets promote alterations to recommended macronutrient ratios to promote health. This review will outline the ways in which specific macro- and micronutrients impact the gut microbiome and host health.

## Introduction

The gut microbiome is the microbial ecosystem comprised of the many different organisms residing in the gastrointestinal tract. Two phyla, Bacteroidetes and Firmicutes, dominate the intestinal environment (Eckburg et al., 2005). Though the gut microbiome is highly dynamic, evidence suggests that we can only alter 30 to 40% of our adult gastrointestinal microbiome; the other 60 to 70% is likely controlled by factors such as genetics, epigenetics, and maternal factors such as maternal health, diet, and breastfeeding (Kashtanova et al., 2016). Many of the over 5 million collective genes of the gut microbiome encode biosynthetic enzymes, glycosidases, and proteases (Sommer and Bäckhed, 2013). The gut microbiome contributes to a wide range of functions vital to the host including immune regulation, organ development, host metabolism, behavior (Sommer and Bäckhed, 2013), and maintenance of the structural integrity of the intestinal mucosa (Jandhyala et al. 2015). A dysbiotic gut microbiome can contribute to health problems and is often characterized by reduced diversity, increased pathobionts, or microbial overgrowth. The intestinal microbiota is thought to play a role not only in inflammatory conditions associated with diet such as inflammatory bowel disease (IBD), obesity, diabetes (Clarke et al., 2014), and cardiovascular disease (Sandoval and Seeley, 2010), but also in other health-related conditions like asthma (Clarke et al., 2014), autism (Vuong and Hsiao, 2017), epilepsy (Dahlin and Prast-Nielsen, 2019), depression, and schizophrenia (Robertson et al., 2017). Research indicates that the gut microbiome is a contributing factor in certain phenotypic traits and conditions (O’Hara and Shanahan, 2006).

Healthy microbiomes can influence the growth of undesirable organisms in a range of environments, intestinal and otherwise. For example, the wooden vats used for making Protected Designation of Origin (PDO) Salers cheese are not sterilized and contain biofilms that are protective against pathogens. Even when milk is inoculated with *Listeria monocytogenes* or *Staphylococcus aureus*, these organisms are found neither on the wooden surfaces nor in the resulting cheeses (Didienne et al., 2012). This example and many others demonstrate that promoting the growth of beneficial organisms is an effective method to prevent the growth of pathogens. These ideas have been implemented in mainstream therapies and in food safety (Niederwerder, 2018). For example, fecal transplants on young chicks are effective in reducing colonization by pathogens like *Salmonella* in poultry by establishing *Salmonella*-free intestinal microbiomes (Snoeyenbos et al., 1982). Not only does early institution of a healthy microbiome prevent overgrowth of pathobionts already present in the gut as well as pathogenic infections in chickens, a healthy gut microbiome can help prevent dysbiosis and pathogenic infection in humans (Kamada et al., 2013). A dysbiotic gut environment is less resistant to infection as pathogens can readily outcompete commensal organisms due to their resistance against host defenses. A more diverse diet results in a more diverse intestinal microbiome (Heiman and Greenway, 2016), which can help prevent certain dysbioses that are exploited by intestinal pathogens and lead to infection. In addition to protecting against infection or overgrowth, a healthy gut microbiota also acts as a barrier that modulates host interactions with xenobiotics (Gaulke et al., 2018).

Dietary components can be divided into three macronutrients—carbohydrates, proteins, and fats, and two types of micronutrients—vitamins and minerals. Macronutrients are required for energy production and anabolism while micronutrients are typically enzymatic cofactors in biochemical reactions. Microbes require nutrients just as humans and animals do, however they are unique in that they both use and produce vitamins. Therefore, microorganisms have a complex relationship with intestinal vitamin levels. Dietary macronutrient ratios and food preparation methods can also influence the gut microbiome and impact the metabolic profile of the gut microbiota (Maier et al., 2017; Shen et al., 2010). The traditional diets of cultures across the world vary in macronutrient ratios. Further, many alternative diets promote altered macronutrient ratios as a means to lose weight or promote health. Therefore, the impact of dietary macro- and micronutrient composition on the intestinal microbiota is highly relevant.

## Macronutrients

### Carbohydrates

The capacity of the intestinal microbiota to metabolize carbohydrates is potentially several thousand times our own genome-encoded ability (Sonnenburg and Sonnenburg, 2014). Carbohydrates can be classified as mono-, di-, oligo-, or polysaccharides. Because only monosaccharides can be taken up through the intestinal wall, larger molecules must first be broken down into their basic subunits prior to absorption (Huber et al., 2017). Sugar is rapidly absorbed in the small intestine where microbial genes related to carbohydrate metabolism are overrepresented compared to the distal colon (Zoetendal et al., 2012). Carbohydrates can be divided into three basic groups that are of nutritional importance: starch, fiber, and simple sugar. Starch is produced by plants as their primary food reserve. Starches are the only human-digestible polysaccharides, as humans lack the glycosidases necessary to cleave the β-1,4 glycosidic bonds found in other polysaccharides. Fiber consists of non-digestible oligo- and polysaccharides, as well as some forms of starch referred to as resistant starch (Huber et al., 2017).

### Fiber

Fiber can be grouped into soluble and insoluble fibers, with soluble fibers conferring health benefits like increased intestinal and fecal bulk, decreased transit time, and decreased serum cholesterol (Huber et al., 2017). As fiber is undigestible by the human gastrointestinal tract, it remains relatively intact as it enters the colon where it is accessible to the intestinal microbiota. A low fiber diet not only affects the gut microbiota of the individual consuming it, but can also reduce the microbial diversity of offspring in a way that is irreversible by fiber alone over only a few generations (Sonnenburg et al., 2016). Whole grains are an example of a high fiber food that contains a variety of microbially accessible compounds, such as beta-glucans and arabinoxylans (Gong et al., 2018). Consumption of whole-grain products can increase *Bifidobacterium*, *Lactobacillus*, *Enterococcus*, *Blautia*, and *Roseburia*, increase the Firmicutes:Bacteroidetes ratio, and decrease *Bacteroides* (Graf et al., 2015). Thirty-nine out of 42 studies also found that cereal fibers increased microbial diversity, abundance, or both (Jefferson and Adolphus, 2019).

Whole grain oat flour increased levels of *Alcaligenaceae*, which can metabolize oestradiol in vitro and may promote the reabsorption of cholesterol (Vitaglione et al., 2019). Some research indicates that most of the impact of grains on the gut microbiome is attributable to phenolic compounds, beta-glucans, and arabinoxylans (Gong et al., 2018), though one paper reported that neither beta-glucans nor polyphenols were responsible for the bifidogenic effect associated with oat consumption (Kristek et al., 2019). Different beta-glucans have various effects on the gut microbiome. Low molecular weight beta-glucans did not alter the microbiomes of human subjects while high molecular weight beta-glucans decreased the Firmicutes:Bacteroidetes ratio, with increases in *Bacteroides* and *Prevotella*, and decreases in *Dorea* (Wang et al., 2016). The model organism influences the observed microbial impact as well. Oat beta-glucan increased *Lactobacillus* and *Bifidobacterium* and decreased *Enterobacteriaceae* in rats (Shen et al., 2012), whereas in pigs it increased *Lactobacillus*, *Streptococcus*, *Enterococcus*, *Clostridium* clusters I and XIVa, certain members of *Bacteroides*-*Prevotella*-*Porphyromonas*, and *Enterobacteriaceae* (Metzler-Zebeli et al., 2011).

Arabinoxylans are the most abundant non-starch polysaccharides present in cereal grains and contribute significantly to gut health (Mendis et al., 2016). Arabinoxylans have been associated with increased *Bifidobacterium* (specifically *Bifidobacterium animalis* ssp. *lactis*), *Prevotella*, *Faecalibacterium prausnitzii*, and *Lactobacillus* as well as decreased *Escherichia coli*, *Streptococcus*, *Staphylococcus*, *Lactobacillus*, *Clostridium histolyticum* I and II, and *Enterococcus* (Gong et al., 2018). Long-chain arabinoxylans also promote the growth of *Bifidobacterium longum* with a concurrent increase in propionate levels (Van den Abbeele et al., 2013) and have been associated with immunomodulatory properties in a variety of organisms (Mendis et al., 2016).

While most starch is digestible, resistant starch (RS) is non-digestible and is therefore classified as a fiber. Resistant starch is traditionally categorized into four groups: RS1 is physically inaccessible, RS2 is ungelatinized raw starch, R3 is retrograded starch, and RS4 is chemically modified resistant starch (Kashantova et al., 2016). *Eubacterium rectale*, *Ruminococcus bromii*, and *Bifidobacterium thetaiotamicron* and *adolescentis* are important organisms in the degradation of RS (Ze et al., 2012). *Ruminococcus bromii* and *B. adolescentis* are better able to ferment RS2 and RS3 than *E. rectale* or *B. thetaiotamicron*, however in co-culture *R. bromii* stimulated the ability of the other three organisms to degrade these compounds. Further, addition of *R. bromii* to fecal microbiomes devoid of *R. bromii* or related organisms showed increased ability to ferment RS3 compared to unsupplemented samples (Ze et al., 2012). Feeding a diet containing RS in humans significantly increases the presence of *R. bromii* and closely related organisms, further supporting their importance in RS metabolism (Abell et al., 2008). Martínez et al. (2010) confirmed the increases in *R. bromii* and *E. rectale* seen with RS2 and found that the effects of RS2 differed from those seen with RS4 in human subjects. Type 4 residual starch promoted the growth of Actinobacteria and Bacteroidetes but led to a decreased presence of Firmicutes while RS2 did not show phylum-level changes. At the species level, RS4 promoted the growth of *B. adolescentis* and *Parabacteroides distasonis* (Martínez et al., 2010). Resistant starch is particularly important, as its fermentation results in higher levels of butyric acid compared to the fermentation of other fibers (Huber et al., 2017).

Butyrate is important as it is used by colonocytes as an energy source, promotes barrier function, and reduces inflammation (Chen et al., 2017). *Prevotella*-rich microbiota produces higher levels of SCFAs through the metabolism of cereal grain fibers than microbiota rich in *Bacteroides*, and different organisms produce various levels and ratios of SCFAs from the same carbohydrate substrate in vitro assay (Chen et al., 2017). Therefore, the existing microbiota will influence how a substrate is metabolized. Both the fiber source and the existing microbiota impact how the microbiota will change in response to fiber consumption. Administration of three different fibers to microbiota dominated by *Prevotella* promoted the growth of the same *Prevotella* operational taxonomic unit (OTU) and resulted in similar SCFA profiles; however, administration of these three fibers to microbiota dominated by *Bacteroides* promoted different OTUs and resulted in different SCFA profiles (Chen et al., 2017).

### Protein

A portion of dietary amino acids are catabolized in the small intestine where they are the primary energy source for enterocytes (Wu, 1998; Zoetendal et al., 2012). Evidence suggests that amino acid availability may be the limiting factor for microbial growth in the small intestine, stimulating microbial de novo synthesis (Zoetendal et al., 2012) and allowing amino acid exchange between the microbiota and their host to run in both directions (Davila et al., 2013; Zoetendal et al., 2012). Though amino acids can be taken up by the small intestine, it is generally accepted that they cannot be absorbed by the colon except for a short period after birth (Darragh et al., 1994). Many microorganisms are able to metabolize proteins, peptides and amino acids, with several being either preferential or obligate fermenters. Key proteolytic organisms in the colon include *Bacteroides*, *Clostridium*, *Propionibacterium*, *Fusobacterium*, *Streptococcus*, and *Lactobacillus* (Davila et al., 2013). Microorganisms differ in their abilities to metabolize proteins. *Bacteroides vulgatus*, *Bacteroides distasonis*, and *Bacteroides ovatus* secrete more proteases than other members of *Bacteroides*. These proteases have similar properties to those secreted by the host (Riepe et al., 1980). Microbial composition and proteolytic activity in the intestinal tract are important factors in gut health. Bacterial proteases are a possible link between intestinal dysbiosis and inflammatory conditions like IBD (Carroll and Maharshak, 2013). Proteases produced by *Bacteroides* reduced the activity of maltase and sucrase and were suggested to be capable of damaging brush border function in cases of bacterial overgrowth (Riepe et al., 1980). A metalloprotease from *Enterococcus faecalis* can also compromise gut barrier function and contribute to gut inflammation and the development of colitis in mice (Steck et al., 2011).

Some products of protein metabolism, such as nitrosamine precursors and trimethylamine N-oxide (TMAO), can have detrimental effects on host health whereas others may confer health benefits (Kashantova et al., 2016). Colonic fermentation of aromatic amino acids can produce phenylpropanoids which can be beneficial to human health. *Bacteroides thetaiotamicron*, *Bacteroides eggerthii*, *B. ovatus*, *Bacteroides fragilis*, *Parabacteroides distasonis*, *Eubacterium hallii*, and *Clostridium bartlettii* can produce significant levels of phenylacetic acid, 4-hydroxylphenylacetic acid, and indole-3-acetic acid, and are likely the major source of these compounds in the colon (Russell et al., 2013). Though protein is an essential part of the diet, protein metabolites can be toxic and some evidence even links red meat to cardiovascular disease and colorectal cancer (Zhu et al., 2015).

A high-protein, low-carbohydrate diet in obese men was associated with decreased SCFAs, increased branched chain fatty acids, a large drop in *Bacteroides*, and a reduction in the major butyrate-producing *Roseburia*/*Eubacterium rectale* group (Russell et al., 2011). Though there was no overall change in *Lachnospiraceae* or the second major group of butyrate-producing organisms related to *F. prausnitzii*, butyrate production was reduced under a high-protein diet (Russell et al., 2011). The drop in *Bacteroides* is counterintuitive, given that they have previously been listed as effective protein metabolizers. Mice fed a protein-deficient diet possessed microbiomes more similar to just-weaned mice than to mice of the same age fed a normal amount of protein and had increased excretion of metabolites, including the microbial metabolite choline and microbe-host cometabolites 3-indoxysulfate, *m*-hydroxyphenylpropionylsulfate, hippurate, and TMAO. Further, certain OTUs were associated with the excretion of specific metabolites, particularly *Akkermansia muciniphila* which was correlated with *N*-phenylacetylglycine, 3-indoxysulfate and trimethylamine, among other things (Mayneris-Perxachs et al., 2016). In contrast to the previous studies that found microbial changes associated with various levels of protein intake, Beaumont et al. (2017) found that a high-protein diet did not affect the microbiome of humans but did alter host gene expression and cause a shift in microbial metabolism toward amino acid degradation that varied with protein source.

Protein sources can be divided into meat, dairy, and plant-based sources. Dairy is often grouped either with meat to make plant-based and animal-based groupings, or with plant-based sources to make meat and non-meat groupings. In rats, meat-based sources lead to increased Firmicutes and fewer Bacteroidetes than non-meat proteins, with white meat having the highest level of *Lactobacillus* and an increased Firmicutes:Bacteroidetes ratio compared to other proteins (Zhu et al., 2015). Chicken is associated with elevated levels of *Actinobacteria* in rats and increased *Bifidobacterium*, and *Bacteroides* in vitro (Shen et al., 2010; Zhu et al., 2015). Red meat diets were associated with elevated levels of *Ruminococcaceae* and *Lactobacillaceae*, and beef increased Proteobacteria and *Oscillibacter* and decreased *C. perfringens* and *C. histolyticum* relative to chicken or fish after 30 h of fermentation (Shen et al., 2010; Zhu et al., 2015). Butteiger et al. (2016) compared the effects of milk protein isolate, soy protein isolate, and soy protein concentrate in hamsters and found that soy protein leads to higher microbial diversity and a shift in microbial metabolism. It was concluded that alterations in the microbiome may be the cause of the lipid-lowering properties associated with soy (Butteiger et al., 2016). Soy, casein, and fish meal fed to rats did not induce microbial changes at the phylum level (An et al., 2014) At the family level, soy induced the highest levels of *Ruminococcaceae* and the lowest levels of *Lactobacillaceae*, and *Bacteroidaceae* was only present with fish meal (An et al., 2014). *Lactobacillus* was present with all three diets, however species varied based on which protein was fed; *Lactobacillus intestinalis* was present with casein, *Lactobacillus reuteri* was present with soy, and *Lactobacillus murinis* was present in those fed fish meal (An et al., 2014). Soy has also been found to increase Bacteroidetes in rats, and soy and casein both lead to increased levels of *Lachnospiraceae* (Zhu et al., 2015). While Butteiger et al. (2016) found that soy increased microbial diversity, Zhu et al. (2015) found that meat consumption increased microbial diversity compared to soy, concluding that animal-based proteins may create a better microbial composition than soy. Further, they conclude that meat proteins create a more balanced gut composition than non-meat proteins (Zhu et al., 2015). Discrepancies between the two studies could be due to species differences of hamsters versus rats or may be attributable to the formulation of the non-protein aspect of the diets. Regardless, the opposing conclusions warrant further investigation to determine how these dietary proteins may influence human health.

### Fat

A high-fat diet can alter the murine microbiome independent of obesity (Hildebrandt et al., 2009), and an altered microbiome characterized by a drop in Bacteroidetes and an increase in Firmicutes was linked with obesity in mice (Ley et al., 2005). Hildebrandt et al. (2009) found that Bacteroidetes abundance decreased upon administration of a high-fat diet irrespective of genetic predisposition to obesity and also noted a positive correlation between Actinobacteria and fat consumption (Hildebrandt et al., 2009). Krill oil increased microbial diversity compared to the reduced diversity created by a high-sugar, high-fat diet and helped to normalize the microbiota of mice fed a Western-type diet (Lu et al., 2017). From a microbial perspective, long-term dairy and saturated fat intake led to significant increases in *B. wadsworthia* and a high-fat, animal-based diet showed increases in microbial DNA and RNA encoding sulfite reductases indicative of an elevated presence of *B. wadsworthia* in humans (David et al., 2013). A diet high in saturated fats creates an environment that promotes the expansion of this sulphite-reducing pathobiont and favors the onset of colitis in susceptible mice (Devkota et al., 2012). Omega-3 fish oil, on the other hand, appears to inhibit the growth of *B. wadsworthia* by inducing changes in the bile acid composition of mice (Devkota and Chang, 2015).

### Bile acid metabolism

There is heavy interplay between diet, microbes, and bile metabolism. The enzymes produced by the intestinal microbiota are as important in bile acid metabolism as those encoded by the host’s own genome (Long et al., 2017). In general, microbial interactions with bile acids increase the diversity and overall hydrophobicity of the bile acid pool, facilitating fecal elimination (Degirolamo et al., 2014). Microbes regulate both the synthesis and metabolism of bile acids, which in turn mold the intestinal microbial community by inhibiting the growth of bile-sensitive bacteria and promoting the growth of bile metabolizing organisms like *B. wadsworthia* (David et al., 2013; Devkota et al., 2012). Bile acids regulate the gut microbiota both through the immune system by inducing the transcription of antimicrobial agents such as iNOS and IL-18 as well as through the direct antimicrobial effects of their detergent-like properties (Inagaki et al., 2006). High-fat diets in mice led to increased levels of enteric deoxycholic acid (DCA), a secondary bile acid that may be linked to obesity and liver cancer, through modulation of the gut microbiota (Yoshimoto et al., 2013). Increased DCA may inhibit certain members of Bacteroidetes and Firmicutes. Animal-based diets in humans result in increased fecal DCA and increased expression of genes encoding bile salt hydrolases, which break down bile acids into DCA (David et al., 2013). Organisms like Actinobacteria, Proteobacteria, Firmicutes, and Bacteroidetes possess hydroxysteroid dehydrogenases (HSDHs) and are able to generate oxo- or keto-bile acids in a reversible reaction that can result in epimerization (Wahlström et al., 2016). These iso-bile acids are found abundantly in the colon and feces, as well as in smaller levels in serum and urine, but are not found in the bile pool (Shefer et al., 1982; Wahlström et al., 2016). They may also be regulators of both the host metabolism and the gut microbial composition, as iso-DCA has been shown to favor the growth of *Bacteroides* in rats (Wahlström et al., 2016). Bacteria associated with the production of iso-bile acids include *Eubacterium lentum*, *C. perfringens*, and *Ruminococcus gnavus* (Wahlström et al., 2016), the last of which may be linked to IBD and lupus (Azzouz et al., 2019; Hall et al., 2017). Bile acid composition is therefore important not only in microbial community structure but also plays a significant role in host health, and there is a tight relationship between the type and level of fat consumed, bile acid makeup, and the gut microbiota.

### Unsaturated fatty acids

Omega-3 and omega-6 fatty acids are polyunsaturated fatty acids that are an essential part of the diet. Omega-6 fatty acids upregulate inflammation as they are precursors to pro-inflammatory signaling molecules, while omega-3 fatty acids compete with this pathway and reduce inflammation (Robertson et al., 2018). Offspring of mice fed diets high in omega-3 displayed slightly higher levels of the anti-inflammatory cytokine IL-10, with reductions in peanut allergies but worsened reactions in certain bacterial infections (Myles et al., 2014). The traditional ratio of omega-6:omega-3 consumed was likely close to 1:1, however the current ratio consumed in the Western diet is 10–50:1. The imbalance in this ratio may be a source of health problems, such as the metabolic endotoxemia and slight systemic inflammation associated with omega-6 that are mitigated by omega-3 (Kaliannan et al., 2015; Robertson et al., 2018). The effect of omega-3 on the gut microbiome has been extensively studied. Omega-3 induced changes in the gut microbial composition of mice which lead to decreased LPS production and intestinal permeability, both of which are increased in certain disease states (Kaliannan et al., 2015; Robertson et al., 2017).

In humans, omega-3 fatty acids and docosahexaenoic acid (DHA) levels correlated with alpha diversity of microbiome and increased *Lachnospiraceae* (Menni et al., 2017), and supplementation with omega-3 increased the butyrate-producing genera *Bifidobacterium*, *Lachnospira*, *Roseburia*, and *Lactobacillus* (Watson et al., 2018). In a murine model, maternal omega-3 supplementation reduced *Lachnospiraceae*, *Anaerotruncus*, and *Roseburia* and increased *Blautia*, *Oscillibacter*, *Clostridiales*, *Robinsoniella*, *Lactococcus*, and *Eubacterium* in offspring (Myles et al., 2014). Omega-3 fatty acid deficient mice displayed an elevated Firmicutes:Bacteroidetes ratio, while omega-3 supplemented mice had increased levels of *Lactobacillus* and *Bifidobacterium* (Robertson et al., 2017). Both human and animal studies have determined that omega-3 fatty acids affect the gut-brain axis through alterations in the gut microbiome. Omega-3 fatty acid deficiency in early life has been related to impaired brain function, including psychomotor activity, cognition, attention, and vision, as well as psychological disorders such as depression, schizophrenia, and dementia (Robertson et al., 2017).

As with other aspects of diet, maternal consumption of omega-3 fatty acids influences the microbiome and disease states of her offspring later in life. These influences are apparent in several murine studies. Maternal fatty acid consumption during lactation had larger impacts on offspring than consumption during pregnancy (Robertson et al., 2018). Changes in dietary omega-3 fatty acids in the mother changed the levels of omega-3s in the mother’s milk, and therefore in the offspring’s diet (Robertson et al., 2017). Additionally, maternal fatty acid compositions were found to be transferrable to male, but not female, offspring (Robertson et al., 2018). Increased omega-3 fatty acid production correlated with reduced weight-gain and improved metabolism in offspring while reduced maternal omega-3s were linked to significant reductions in epsilon-proteobacteria, *Bacteroides*, and *Akkermansia*, as well as an increase in *Clostridia*, indicating that microbial restructuring could be contributing to the phenotypic changes (Robertson et al., 2018). Myles et al. (2014) found that, compared to mice fed a low-fat diet, the offspring of mice fed high levels of omega-3 fatty acids displayed the reduced Bacteroidetes and increased ratio of Firmicutes:Bacteroidetes frequently associated with obesity, however they were not obese and did not have the increased inflammation typical of Western diets high in omega-6 fatty acids (Myles et al., 2014).

Conjugated linoleic acids (CLA), which are present mainly in meat and dairy, are a group of omega-6 fatty acids that can reverse the problems associated with a high-fat diet and promote the growth of Bacteroidetes, Prevotella, and *A. muciniphila* in mice (Chaplin et al., 2015). *Trans*-10, *cis*-12 CLA, which is found in dairy products, meat from ruminants, and is produced by organisms such as *Lactobacillus plantarum* PL62, demonstrates antiobesity effects but appears to induce hepatic steatosis and hyperinsulinemia, both of which are observed in diabetic or obese individuals (Lee et al., 2007; Marques et al., 2015). In mice, *trans*-10, *cis*-12 CLA reduced the Firmicutes:Bacteroidetes ratio and decreased levels of Desulfovibrionaceae, Lachnospiraceae, Peptococcaceae, and Clostridiales Family XIII. Incertae Sedis while increasing Porphyromonadaceae (Marques et al., 2015).

The impact of the unsaturated:saturated fatty acid ratio on the gut microbiome and human health has been investigated through the use of various plant and animal oils. De Wit et al. (2012) focused on the different effects associated with low, roughly equal, and high ratios of polyunsaturated to saturated fatty acids in high-fat diets. High-fat palm oil, olive oil, and safflower oil diets were compared to a low-fat palm oil diet in mice. High-fat palm oil and high-fat olive oil diets led to increase obesity despite a lack of change in the gut microbiota between the high fat olive oil diet and the control. The levels of *Deferribacteres* varied with each diet, but the microbiota associated with the control, olive oil, and safflower oil diets did not otherwise differ. A high-fat palm oil diet reduced microbial diversity and increased the Firmicutes:Bacteroidetes ratio, especially with respect to *Clostridium* clusters XI, XVII, and XVIII (de Wit et al., 2012). Hidalgo et al. (2014) found an increased level of Bacteroidetes in mice fed olive oil compared to those fed palm oil (Hidalgo et al., 2014). The fatty acid profile of olive oil can vary widely, as can the phenolic content. Extra virgin and refined olive oil exhibited the microbiota more similarly to each other than to butter, which showed an elevated Firmicutes:Bacteroidetes ratio and a significant increase in Lactobacillaceae. The microbial profile associated with refined olive oil was between that of butter and extra virgin olive oil, which is interesting given that the only difference between the olive oils used in the study was the phenolic content (Hidalgo et al., 2014).

## Micronutrients and heavy metals

### Vitamins and minerals

Micronutrients are compounds that are vital in small amounts for proper development and function but in large doses often become toxic. Deficiency in the vitamin A metabolite retinoic acid impairs immunity while excess can lead to inflammatory disorders (Lee and Ko, 2016). Micronutrients can be classified as vitamins, which are organic, and minerals, which are inorganic. While macronutrients are broken down and used in energy production and anabolism, micronutrients largely function as enzymatic cofactors in biochemical reactions. Prokaryotes require micronutrients just as eukaryotes do, however unlike animals and humans which need to obtain both vitamins and minerals through their diet, prokaryotes are able to synthesize certain vitamins. There is debate regarding the bioavailability of vitamins produced by the gut microbiota, and the answer likely depends on which region of the gut various compounds are synthesized in. Nearly all vitamin absorption takes place in the small intestine, however some may occur in the colon as well (Biesalski, 2016). Thus, the gut microbiota both influence and are influenced by levels of micronutrients.

### Vitamins

Vitamins have a significant impact on the immune system and intestinal inflammatory diseases are often associated with vitamin deficiency (Biesalski, 2016; Jin et al., 2015). Vitamin A, for example, was found to be anti-viral against norovirus infection (Lee and Ko, 2016) and vitamin D is known to benefit immune and cardiovascular function. Vitamin A had a larger impact on gut microbial community structure and gene expression than folate, iron, or zinc (Hibberd et al., 2017). Vitamin D deficiency and vitamin D receptor (VDR) downregulation are associated with inflammatory and autoimmune diseases (Bashir et al., 2016; Jin et al., 2015). Norovirus-infected mice supplemented with retinoic acid exhibited increased *Lactobacillus*, whereas norovirus infection alone resulted in decreased Lactobacillaceae populations. *Lactobacillus* and retinoic acid each were found to have independent anti-viral activity against norovirus (Lee and Ko, 2016). Supplementation with retinoic acid in the absence of norovirus infection increased *Allobaculum*, *Aggregatibacter*, *Bifidobacterium*, *Dialister*, and *Enhydrobacter* in mice (Lee and Ko, 2016).

In humans, vitamin D supplementation decreased the relative abundance of gammaproteobacteria such as *Pseudomonas*, *Escherichia*, and *Shigella* and increased bacterial richness in the upper gastrointestinal tract but did not result in microbial changes downstream of the terminal ileum (Bashir et al., 2016). Vitamin D receptors also regulate antimicrobial peptide expression which can impact the microbiome, and microbial byproducts such as secondary bile acids may be able to activate VDRs (Clark and Mach, 2016; Jin et al., 2015). VDR knockout mice had lower levels of *Lactobacillus* and increased *Clostridium* and *Bacteroides* in their feces and decreased *Alistipes* and *Odoribacter* with increased *Eggerthella* in their ceca (Jin et al., 2015). Another mouse study found that vitamin D increases beneficial microorganisms, including Lactobacillaceae, and protects against induced colitis (Ooi et al., 2013). It is also hypothesized that supplying different ratios of corrinoids, the family of compounds to which vitamin B_12_ belongs, can shape the microbiome as different microbes require specific corrinoids (Degnan et al., 2014), though this remains to be tested.

Intestinal microbes can also produce many vitamins including vitamin K_2_ (menaquinone), B_12_ (cobalamin), and biotin. Different organisms are capable of producing specific vitamins—for example, certain members of *Bifidobacteria* can produce folate (LeBlanc et al., 2013). The bioavailability of microbially produced vitamin K is questionable as absorption occurs in the small intestine while production presumably occurs in the large intestine (Biesalski, 2016). To be host-available, production of a compound should occur at or upstream of the absorption site. Zoetendal et al. (2012) investigated the small intestinal microbiota by use of ileostomists, individuals who have had their colon removed, and discovered increased synthesis of cobalamin and biotin in ileostomy samples compared to fecal samples. (Zoetendal et al., 2012). Genes related to cobalamin and biotin synthesis were associated mostly with Proteobacteria but were also linked to Firmicutes and Bacteroidetes (Zoetendal et al., 2012). Since most biotin absorption occurs in the small intestine, it was determined that microbes may supply a significant level of biotin to their hosts (Zoetendal et al., 2012). Some water-soluble vitamins, including biotin, can also be taken up by colonocytes (Biesalski, 2016). Conversely, microbially derived cobalamin is not likely shared with human hosts and microbes more likely compete with the host for dietary cobalamin (Biesalski, 2016; Degnan et al., 2014).

Most of the microorganisms present in the intestinal tract lack the genes for cobalamin production and therefore require exogenous cobalamin (Degnan et al., 2014). Further, cobalamin produced in the colon where microbial load is highest is not bioavailable as uptake occurs in the small intestine (Degnan et al., 2014). While fewer organisms are present in the small intestine, Zoetendal et al. (2012) found genes related to cobalamin and biotin synthesis to be overrepresented in ileostomy samples (Zoetendal et al., 2012). There is a disconnect between microbiome data and information on vitamin absorption, as the vast majority of vitamin absorption occurs in the small intestine but most microbiome studies are performed on fecal samples. Further studies investigating vitamin production and absorption along the intestinal tract, specifically in the small intestine, are required to understand the bioavailability of microbially-produced vitamins such as menaquinone and cobalamin. Even if the microbially-synthesized vitamins produced inside the intestinal tract are not host-available, microbes can still impact vitamin intake through their application in the food industry. Since different organisms use and produce different levels of vitamins, vitamin levels in fermented foods can be manipulated through selection of starter cultures (LeBlanc et al., 2013). While vitamin levels can be manipulated through the use of specific taxa, mineral content cannot be influenced this way.

### Minerals

Fewer studies are available on the effects of minerals on the gut microbiome. Mineral deficiencies are prevalent throughout the world, particularly in low-resource areas, and can result in pathologies ranging from iron deficiency anemia to various enteropathies (Mayneris-Perxachs et al., 2016; Tang et al., 2017). Mineral excess can also result in gastrointestinal distress, making it important to understand the impact that minerals have on the gut microbiota. Mayneris-Perxachs et al. (2016) found that the microbiomes of zinc-deficient mice were comparable to their well-nourished counterparts (Mayneris-Perxachs et al., 2016). Zinc oxide was found to modulate the microbiome of piglets in a manner similar to antibiotics, possibly explaining why zinc supplementation is effective in preventing and treating diarrhea in piglets (Yu et al., 2017). Antibiotics and zinc both resulted in changes to the non-predominant microbiota of the ileal digesta and colon (Yu et al., 2017). In the ileal digesta, zinc and antibiotic supplementation at a pharmacological level increased Spirochaetes, Tenericutes, Euryarchaeota, Verrucomicrobia, TM7 and Enterobacteriales, and decreased Chlamydiae and Campylobacterales (Yu et al., 2017). Zinc also decreased the amount of antibiotics necessary to induce *Clostridium difficile* infection, a common cause of antibiotic-associated diarrhea and other nosocomial infections (Zackular et al., 2016). Microbial changes included decreased diversity, decreased *Turicibacter* and *Clostridium*, and increased *Enterococcus* and *Clostridium* XI (Zackular et al., 2016). Micronutrient deficiencies such as zinc deficiency can increase susceptibility to the effects of toxin exposure, for example lead or arsenic (Bushnell and Levin, 1983; Gaulke et al., 2018).

Iron deficiency is one of the most prevalent micronutrient deficiencies, however iron supplementation is often associated with adverse effects (Tang et al., 2017). Excessive iron intake can promote the growth of pathogenic strains of organisms such as *Salmonella*, *Shigella*, and *E. coli*, and multiple studies cited by Tang et al. (2017) have found that iron supplementation or fortification can result in unfavorable gut microbiomes, for example with depleted *Bifidobacterium* and increased pathogens, and increased gut inflammation in children and infants (Tang et al., 2017). Tang et al. (2017) found that, while iron supplementation did not cause an increase in *E. coli* and *Shigella*, it did prevent their normal decrease in infants (Tang et al., 2017). Since nutrition can significantly impact the intestinal microbiota and dysbiosis has been correlated with a range of afflictions including psychiatric disorders, Winther et al. (2015) tested the hypothesis that imbalances in the gut-brain axis induced by magnesium deficiency could lead to depressive-like behavior. Mice fed a magnesium deficient diet had changes in their gut microbiome that positively correlated with hippocampal IL-6, and expressed increased depressive-like behavior (Winther et al., 2015). Specific taxa were not investigated, leaving the potential role that micronutrients play in the gut-brain axis largely unexplored. While micronutrients are organic and inorganic compounds essential to proper cell function, organic and inorganic toxicants and contaminants which make their way into the food supply can impede function and be a detriment to human health.

## Heavy metals

Heavy metals are inorganic compounds that exist in the environment and often make their way into the food supply, with levels ranging from low concentrations to above safe limits, as is the case in the vegetables, meat, and fish in a mining city in China (Cheng et al., 2017). This city is not an isolated occurrence; heavy metals such as cadmium, arsenic, lead, and mercury have all been found in marine fish (Bosch et al., 2016). The level of concern varies based on the rates of bioaccumulation in different tissues and the form of the compound present.

### Methylmercury

Methylmercury (MeHg) is the most widely known contaminant of fish and can be a major concern with the consumption of certain species, especially for vulnerable populations including children and women who are pregnant or nursing. Gut microbes can modulate mercury toxicity through methylation and demethylation and may be able to decrease MeHg bioavailability (Lin et al., 2020; Rothenberg et al., 2016). Little difference was seen between the microbiota of human subjects with high stool MeHg compared to low, and any change in diversity was attributed to rare taxa (Rothenberg et al., 2016). Methylmercury treated rats presented with decreased Bacteroidetes and Proteobacteria and increased Firmicutes (Lin et al., 2020). Conversely, methylmercury increased Bacteroidetes and decreased Firmicutes in mice and resulted in genus and species-level changes including increased *Prevotella*, *Odoribacter*, and *Lactobacillus ruminis* (Zhang et al., 2019). In rats, MeHg exposure decreased *Lactobacillaceae*, *Bacteroidaceae*, *Streptococcaceae*, and *Sutterellaceae*, increased *Desulfovibrionaceae*, *Helicobacteraceae*, *Peptococcaceae*, and *Rhodospirillaceae*, and negatively impacted the gut-brain axis (Lin et al., 2020).

### Cadmium

Not all heavy-metal contaminants are as well-known as Methylmercury. Cadmium is a lesser-known threat that impacts the global population (Ba et al., 2017). Many regions have an average weekly cadmium intake at or above tolerable levels. Early life exposure in male mice reduced microbial diversity, altered microbial composition, and contributed to adult adiposity even in cases where the microbiota fully recovered. Microbial effects included increased Bacteroidetes and decreased Firmicutes as well as reduced *Bifidobacterium* and *Prevotella* and increased *Sphingomonas* (Ba et al., 2017). Zhang et al. (2015) also found that cadmium decreases Firmicutes and Gammaproteobacteria, and increases Bacteroidetes (Zhang et al., 2015).

### Lead and arsenic

Similar to cadmium, lead has been linked to body weight gain and obesity (Jin et al., 2017). Microbial changes associated with lead intake include an increased Firmicutes:Bacteroidetes ratio, increased *Desulfovibrionaceae*, *Barnesiella*, and *Clostridium* XIVb, and decreased *Caulobacterales* and *A. muciniphila* (Jin et al., 2017). Arsenic in fish is less of a concern as up to 90% of the arsenic measured in fish is in the less toxic organic form (Bosch et al., 2016), however arsenic is regularly encountered by a large portion of the global population through food and drinking water (Gaulke), the latter of which often contains the more toxic inorganic form (Bosch et al., 2016; Jin et al., 2017). Arsenic has been linked to a decrease in Firmicutes and an increase in Bacteroidetes (Jin et al., 2017). Arsenic exposure and zinc deficiency, which are comorbid in a large portion of the population, have independent impacts on the gut microbiota and together act synergistically (Gaulke et al., 2018). These microbial changes were linked to physiological changes, which is expected as the gut microbiome modulates host interactions with xenobiotics and changing the structure could change host toxin exposure (Gaulke et al., 2018).
