# Correction to: Understanding the effects of dietary components on the gut microbiome and human health

**DOI:** 10.1007/s10068-020-00844-1

**Published:** 2020-12-16

**Authors:** Bryna Rackerby, Hyun Jung Kim, David C. Dallas, Si Hong Park

**Affiliations:** 1 Department of Food Science and Technology, Oregon State University, Corvallis, OR 97331 USA; 2 Korea Food Research Institute, Wanju, Jeollabuk-do 55365 South Korea; 3 School of Biological and Population Health Sciences, Nutrition, Oregon State University, Corvallis, OR 97331 USA

## Correction to: Food Sci Biotechnol (2020) 29(11):1463–1474 10.1007/s10068-020-00811-w

The article “Understanding the effects of dietary components on the gut microbiome and human health”, written by Bryna Rackerby, Hyun Jung Kim, David C. Dallas, Si Hong Park, was originally published Online First without Open Access. After publication in volume 29, issue 11, page 1463–1474 the author decided to opt for Open Choice and to make the article an Open Access publication. Therefore, the copyright of the article has been changed to © The Author(s) 2020 and the article is forthwith distributed under the terms of the Creative Commons Attribution 4.0 International License, which permits use, sharing, adaptation, distribution and reproduction in any medium or format, as long as you give appropriate credit to the original author(s) and the source, provide a link to the Creative Commons licence, and indicate if changes were made. The images or other third party material in this article are included in the article’s Creative Commons licence, unless indicated otherwise in a credit line to the material. If material is not included in the article’s Creative Commons licence and your intended use is not permitted by statutory regulation or exceeds the permitted use, you will need to obtain permission directly from the copyright holder. To view a copy of this licence, visit http://creativecommons.org/licenses/by/4.0.

The original article has been corrected.

